# (p)ppGpp mediates persister formation in *Escherichia coli* during glucose to fatty acid shift

**DOI:** 10.3389/fmicb.2025.1749456

**Published:** 2026-01-16

**Authors:** Ruixue Zhang, Zhengyang Xiao, Neha Namburi, Yinjie Tang, Joshua Yuan, Fuzhong Zhang

**Affiliations:** 1 Department of Energy, Environmental & Chemical Engineering, Washington University in St. Louis, St. Louis, MO, United States; 2 Division of Biological and Biomedical Sciences, Washington University in St. Louis, Saint Louis, MO, United States; 3 Institute of Materials Science and Engineering, Washington University in St. Louis, Saint Louis, MO, United States

**Keywords:** (p)ppGpp, bacterial persistence, nutrient shift, single-cell imaging, stress response

## Abstract

Bacterial persistence contributes to antibiotic failure and recurrent infectious disease, yet the metabolic cues that promote persister formation remain poorly understood. Here we investigated *Escherichia coli* persistence after nutrient downshifts from glucose to various carbons. Compared to shifts to gluconeogenic carbons (pyruvate, malate, succinate, and fumarate), the glucose-to-fatty acid shift induced exceptionally high persister levels, with cells tolerating ampicillin (56%), carbenicillin (22%), and gentamicin (1%) after 24-h treatment. With an RNA-based biosensor and HPLC quantification, we detected up to 4-fold higher guanosine tetra- and penta-phosphate [(p)ppGpp] during the prolonged carbon starvation period post glucose-to-fatty acid shift, whereas (p)ppGpp levels remained low after glucose-to-gluconeogenic carbon shifts due to the shorter lag phase. Shortening the lag phase by pre-exposing cells to fatty acid substantially reduced persistence after the glucose-to-fatty acid shift. Overexpression of acyl-ACP synthase, which acylates free acyl carrier protein and thereby suppresses SpoT-dependent (p)ppGpp synthesis, lowered (p)ppGpp levels and reduced persistence. Furthermore, overexpression of PlsB, a growth-essential enzyme in phospholipid biosynthesis that is inhibited by (p)ppGpp, also reduced persistence. In addition, ^13^C isotope tracing and metabolomic analysis revealed that persisters remain metabolically adaptive, rerouting measurable carbon flux into gluconeogenesis and the pentose phosphate pathway for biomass synthesis. The metabolic remodeling could assist cells to balance redox homeostasis and mitigate oxidative stress. These findings establish the role of (p)ppGpp in nutrient shift persister formation and highlights critical pathways that may be targeted to reduce persistence and improve treatment outcomes against recurrent infections.

## Introduction

Bacterial persister refers to a fraction of isogenic cells who are capable of surviving antibiotic treatment and regrowing once antibiotics are no longer present (Balaban et al., 2004; Lewis, 2007; Fisher et al., 2017). Persisters have been identified across diverse pathogens, including bacteria, fungi, and parasites (Niu et al., 2024; Yuan et al., 2024). The reversible loss of antibiotic susceptibility in persister cells poses a critical clinical challenge, as it drives antibiotic therapy failure, recurrent infections, biofilm formation, and the potential evolution of genetic resistance (Allison et al., 2011a; Brauner et al., 2016; Bakkeren et al., 2020). The global surge in chronic infectious and inflammatory diseases has underscored persisters as a major public health concern (Fisher et al., 2017). Understanding and targeting persister cells represents a promising frontier for overcoming persistent infections, reducing relapse rates, and curbing the escalating crisis of antibiotic resistance.

Unlike antibiotic-resistant mutants, which evade drugs through genetic alterations, persister cells survive through distinct, reversible physiological alterations that allow them to tolerate lethal antibiotic (ABX) concentrations, such as by reducing drug uptake, preventing antibiotic-target interactions, or mitigating downstream lethal effects (Lewis, 2007; Allison et al., 2011a; Fang and Allison, 2023). The elucidation of the molecular mechanisms driving bacterial persister formation is integral to understanding how these cells withstand antibiotic treatment. Extensive research has elucidated multiple molecular mechanisms underlying bacterial persister formation, including metabolic dormancy, DNA damage repair, stress response, cellular communication, efflux pump, and epigenetic modifications (Moyed and Bertrand, 1983; Spoering et al., 2006; Kohanski et al., 2007; Vega et al., 2012; Wu et al., 2012; Kim et al., 2022; Niu et al., 2024; Valencia et al., 2024). The existence of multiple, distinct pathways suggests persister formation is context-dependent and that each persister phenotype may follow its own unique route to persistence (Allison et al., 2011a; Zhang, 2014; Niu et al., 2024).

In natural conditions, bacteria constantly face nutrient fluctuation, with the type, concentration, and availability of substrates vary greatly in the microenvironment (Conway et al., 2004). Such nutrient changes could greatly alter bacterial metabolism, leading to systems level changes that affect many cellular processes, including persistence (Biselli et al., 2020; Gao et al., 2025). Unraveling the metabolic impacts during nutrient shifts may illustrate new persistence formation mechanism, leading to innovative approaches to prevent or eliminate persister cells in changing environments and effective infection control in clinical settings.

Previously, we reported an atypical tri-phasic antibiotic killing kinetics, characterized by a transient tolerance period, a rapid killing phase, and a slower persister killing phase when switching cells from a gluconeogenic carbon source to fatty acid (FA) supplemented with ampicillin (AMP). The transient tolerance right after nutrient shifts is caused by the time needed to accumulate FA degradation enzymes for sufficient carbon catabolism (Hartline et al., 2022). During the subsequent FA catabolism, reactive oxygen species (ROS), likely induced by ampicillin, kill majority of cells (e.g., 99.99% after glycerol to oleic acid with ampicillin shift) through the secondary killing mechanism without cell lysis (Zhang et al., 2024). In contrast, *E. coli* cells after a glucose (GLU) to oleic acid (OA) switch exhibited a high-level persistence to ampicillin (GLU → OA + AMP, 56%) due to the lack of ROS accumulation. Yet it is unclear why cells were not killed by ampicillin nor grow in the presence of FA upon expression of FA degradation enzymes.

In this study, we investigated the persistence frequencies of *E. coli* cells during the nutrient downshift from glucose to various carbon sources. Time-lapse microscopy demonstrated that cells were killed through the ampicillin primary killing mechanism when switching from glucose to pyruvate, succinate, fumarate, and malate, except for the glucose to oleic acid switch. We observed that (p)ppGpp accumulation during the transient tolerance period inhibits cell growth, thereby promoting persistence. Additionally, we identified that (p)ppGpp mediates persister formation by inhibiting glycerol-3-phosphate 1-O-acyltransferase (encoded by *plsB*). The inhibitory process can be mitigated by overexpressing *plsB* or acyl-ACP synthetase (encoded by *aas*), which rebalance acyl-acp thus reducing (p)ppGpp concentration. Our findings confirmed the significant role of (p)ppGpp in regulating persister levels during nutrient downshift to OA and suggest potential therapeutic targets to combat bacterial persistence.

## Materials and methods

### Chemicals and materials

Phusion DNA polymerase, restriction enzymes, and T4 ligase used in plasmid construction were purchased from Thermo Fisher Scientific (Waltham, MA, United States). DNA-spin Plasmid DNA Purification Kit and MEGA quick-spin Total DNA Purification Kit were purchased from iNtRON Biotechnology (Burlington, MA, USA). Antibiotics and inducers were purchased from Gold Biotechnology, Inc. (St. Louis, MO, USA). PpGpp standard was purchased from Jena Biosciences (Jena, German). Other chemicals were purchased from Sigma-Aldrich (St. Louis, MO, USA).

### Bacterial strains, plasmids, and culture conditions

The *E. coli* K-12 strain NCM3722, sourced from the Coli Genetic Stock Center (Yale, USA), was employed as the wild-type strain. All strains and plasmids are listed in Supplementary Tables S1, S2, respectively. *E. coli* MDS42pdu strain was used for cloning. DNA Golden Gate Assembly and BglBrick (Lee et al., 2011) were used for plasmid construction. Primers were designed by Benchling (San Francisco, CA, USA) and synthesized by Integrated DNA Technologies (Coralville, IA, USA).

The ppGpp RNA-based biosensor (pET-28c S2) was purchased from Addgene (plasmid #171920) and transformed into NCM3722 ybhC: T7 RNAP strain, which was made through inserting a T7 RNA polymerase expression cassette into the WT NCM3722 genome. Specifically, T7 RNA polymerase gene, LacI, LacI operator and Int 1 (T7-RNAP) were cloned from *E. coli* BL21(DE3) and was inserted into the ybhC gene site (Ye et al., 2021) of NCM3722 according to the pTarget-pCas homologous recombination protocol as described previously (Jiang et al., 2015, 2017). Colony PCR was used for mutant selection and the DNA fragment sequences were further confirmed by Sanger sequencing (South Plainfield, NJ, USA).

Genes encoding Aas, PlsB, and GFP were overexpressed from plasmids, which were constructed by inserting their PCR amplified DNA sequence into a low-copy number (SC101origin of replication), chloramphenicol resistant plasmid under the control of a LlacO-1 promoter (Lee et al., 2011).

All strains were grown from single colonies and cultivated at 37 °C with 250 rpm of shaking following previous protocols (Hartline et al., 2020, 2022; Zhang et al., 2024). Luria-Bertani (LB) media (10 g NaCl, 10 g Tryptone and 5 g Yeast Extract) was used for cloning and amplifying population from single colony. M9 minimum media [75 mM MOPS at pH 7.4, 2 mM magnesium sulfate, 1 mg/L thiamine hydrochloride, 10 μM iron(II) sulfate, 100 μM calcium chloride, 3 μM ammonium heptamolybdate, 0.4 mM boric acid, 30 μM cobalt(II) chloride, 15 μM copper(II) chloride, 80 μM manganese(II) chloride, and 10 μM zinc sulfate] supplemented with different carbon sources, such as glucose (12 mM), oleic acid (4 mM), malate (18 mM), succinate (18 mM), fumarate (18 mM), or pyruvate (18 mM), was used for persister assay. All cultures were supplemented with appropriate antibiotics (ampicillin, 100 mg/L; kanamycin, 50 mg/L; streptomycin, 100 mg/L; chloramphenicol, 25 μg/mL; carbenicillin, 100 mg/L; gentamicin, 20 mg/L).

### Nutrient shift persistence assay

Nutrient shift protocols were developed following previous methods (Kotte et al., 2014; Hartline et al., 2022; Zhang et al., 2024). In brief, exponentially growing cells cultured in M9 glucose media were harvested by centrifugation (4,500 × *g*, 10 min, 4 °C) and washed three times with ice-cold carbon-free M9 media to remove pre-shift carbon completely. The cells were subsequently resuspended in M9 media, which was supplemented with oleic acid, fumarate, succinate, pyruvate, or malate, respectively, to an initial optical density (OD_600_) of 0.1–0.2. The cell culture was challenged with appropriate antibiotics (ampicillin, 100 mg/L; carbenicillin, 100 mg/L; gentamicin, 20 mg/L) immediately after the nutrient shift. At designated time points post-nutrient shift, 1 mL aliquots were collected, centrifuged (4,500 × *g*, 5 min, 4 °C), and washed four times with ice-cold PBS (pH 7.4) to remove residual ampicillin. Cells were resuspended in PBS, serially diluted, and 5 μL aliquots were plated onto LB agar plates. After overnight incubation at 37 °C, colonies were counted to determine the colony-forming units per milliliter of culture. For strains harboring gene overexpression plasmids, 1 mM IPTG was added to the pre-shift M9 glucose culture at OD600 ≈ 0.1 and maintained throughout the assay. For NCM3722 yhbC:T7 RNAP cells carrying the ppGpp biosensor, 1 mM IPTG was added 2 h before the nutrient shift and maintained throughout the assay.

### Time lapse microscopy of antibiotic killing phase

Cell cultures were pelleted by centrifugation (6,500 × *g*, 4 °C, 5 min), washed, and resuspended in 10 μL of M9 media containing ampicillin and the appropriate carbon source. Next, 1 μL aliquot of the resuspended cells was transferred onto a 1% M9 agarose pad supplemented with ampicillin and the same carbon source, prepared according to Wilmaerts et al. (2022). The agarose pad was then sealed with a coverslip and incubated at 37 °C in a humid chamber (Tokai Hit, Incubation Systems for Microscopes, Japan) and imaged using a Nikon Eclipse Ti microscope (Nikon Instruments Inc., USA) equipped with an EMCCD camera (Photometrics Inc., Huntington Beach, CA, USA) and a 100×, NA 1.40, oil-immersion phase-contrast objective lens. An X-Cite 120 LED was used as the light source. Time-lapse images were acquired every 5 min by an automated scanning function of the microscope with a built-in perfect focus system.

### Single cell (p)ppGpp imaging and analysis

To quantify ppGpp using the biosensor, 200 μM of DFHBI-1 T was added to 1 mL of cell culture, followed by incubation in the dark at room temperature for 30 min. Cells were then pelleted by centrifugation (6,500 × *g*, 5 min) and resuspended in 10 μL 1x PBS. A 1 μL aliquot of the resuspended cells was used for imaging. Images were acquired using microscope as mentioned above with a FITC (λ_ex_ = 480 ± 30 nm, λ_em_ = 510 ± 10 nm, Nikon Instruments Inc., USA) filter cube for spectral separation. More than 300 single cells per sample were collected and analyzed using the Nikon NIS-Elements software package following previous methods (Han and Zhang, 2020).

### (p)ppGpp quantification with HPLC

ppGpp quantification was performed as described before (Radzikowski et al., 2016) with slight modifications. At least 2 × 10^10^ cells were harvested by centrifugation (10,000 × *g*, 4 °C, 1 min), and cell pellet was immediately resuspended in 3 mL of 1 M ice-cold formic acid. Samples were then incubated at 0 °C for 60 min with intermittent vertexing. Following incubation, the suspension was centrifuged (21,000 *g*, 4 °C, 1 min) and the supernatant was transferred to a new tube and flash-frozen in liquid nitrogen. Water and formic acid were removed by freeze-drying overnight. The dried metabolites were resuspended in 0.13 mL of 0.1 M formic acid by vertexing and ultrasonication, then filtered by centrifugation (21,000 × *g*, 4 °C, 1 min) through a Spin-X centrifuge filter (Corning, 0.22-μm nylon membrane, 4,000 *g*). Samples were analyzed using an Agilent 1,260 HPLC system equipped with a multi-wavelength detector (MWD) monitoring absorbance at 260 nm. A PL-SAX anion exchange column (10 μm, 150 × 4.6 mm, 1,000Å, Agilent) was used at a column temperature of 60 °C. A 35-minuate linear gradient of two solvents was applied, changing from Buffer A (0.01 M K_2_HPO_4_, pH 2.6) to Buffer B (0.5 M K_2_HPO_4_, pH 3.5) in 30 min, using a flow rate of 1 mL/min. Peak areas were quantified using a ppGpp standard curve prepared in 0.1 M formic acid. Intracellular concentrations were calculated based on the number of cells harvested and cell volume (Volkmer and Heinemann, 2011).

### ^13^C-labeling of proteinogenic amino acid

Following a nutrient shift from ^12^C-glucose to ampicillin-containing ^13^C-Oleic acid, samples were collected at 5, 9, and 24 h post shift. At least 2 × 10^10^ cells were added to pre-chilled 0.9% (wt/vol) NaCl solution (1:1 volume ratio) to quench the metabolism. Cells were then pelleted by centrifugation (8,000 *g* at 4 °C, 10 min), and the pellet was resuspended in 1.5 mL of 6 M HCl and hydrolyzed at 100 °C for 20 h. The hydrolysates were then transferred to microcentrifuge tubes, and debris was removed by centrifugation (20,000 × *g*, 5 min). Amino acids were derivatized the by adding 300 μL of tetrahydrofuran (THF) and TBDMS (1:1, vol/vol), followed by incubation at 70 °C for 1 h. Samples were analyzed with GC–MS after removing the debris with centrifugation and data was processed as described previously (Hollinshead et al., 2019; Sun et al., 2025).

### Relative metabolite abundance analysis

After performing a GLU → OA + AMP shift, samples were collected at 5, 9, and 24 h post shift. Cells exponentially growing in M9 ^13^C-glucose media were collected as reference. At least 2 × 10^10^ cells were collected and added to pre-chilled 0.9% (wt/vol) NaCl solution (1:1, vol/vol) to quench metabolism. Cell pellet was then collected by centrifugation at 5,000 × *g*, 0 °C for 3 min, and then resuspended in 750 μL methanol-chloroform solution (volume ratio 1:1). Experimental samples after the nutrient shift were combined with reference sample (cells exponentially growing in M9 ^13^C-glucose) and then incubated at 4 °C with shaking at 250 rpm for 4 h. After incubation, 500 μL of ice-cold HPLC grade water was added, and the mixture was vortexed for 2 min. The mixture was then centrifuged at 600 × *g* for 8 min at −10 °C to allow phase separation. The aqueous phase was carefully transferred to a clean tube and filtered using a 3 kDa cut-off centrifugal filter (21,000 × *g*, −10 °C, 90 min). The filtrate was frozen in liquid nitrogen and freeze-dried. Dried metabolites were resuspended in 100 μL of methanol-chloroform (1:1, vol/vol) and analyzed with LC–MS as previously described (Hollinshead et al., 2016, 2019).

### Statistical analysis

Statistical analysis was conducted using GraphPad Prism. The data utilized originate from at least three biological replicates, where applicable. Statistical outcomes are denoted by asterisks (*p < 0.05, **p < 0.01, ***p < 0.001, ****p < 0.0001), indicating significant results, or by the absence of asterisks (non-significant).

## Results

### Nutrient downshift from glucose to oleic acid uniquely promotes persistence

**Figure 1:**
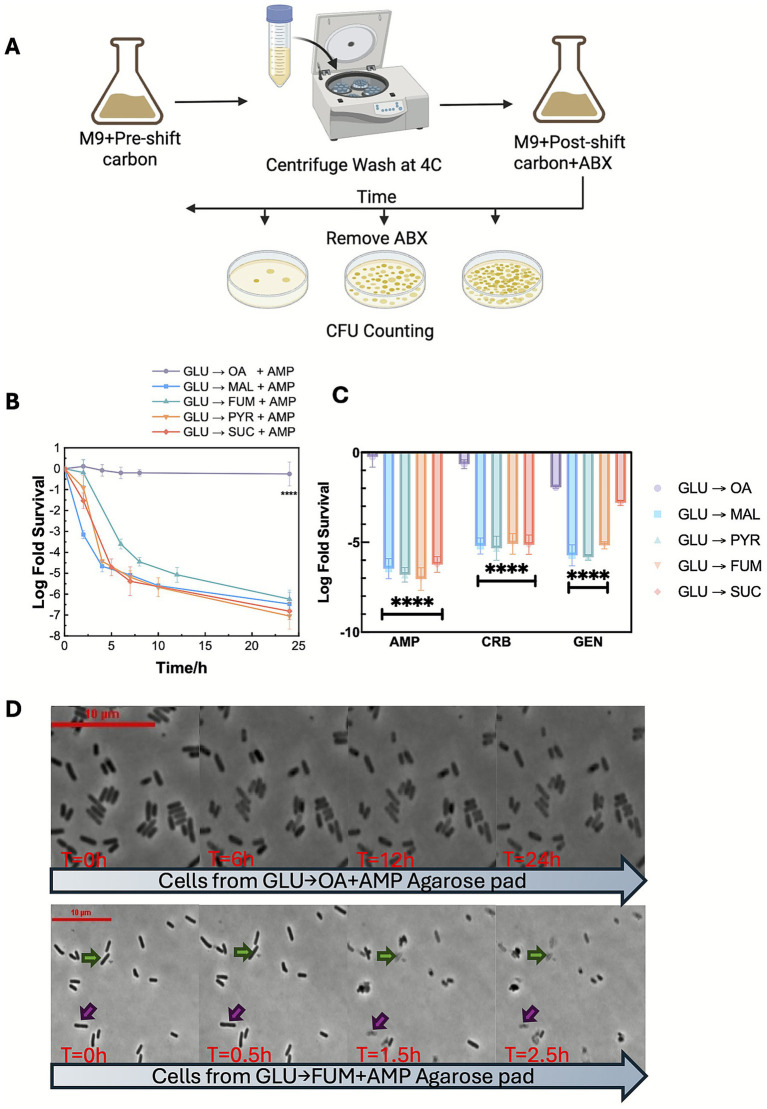
Nutrient downshift from glucose to oleic acid promotes persistence. **(A)** Illustration of nutrient shift persister assay experimental protocol. **(B)** Time–kill curves of *Escherichia coli* after nutrient downshift from M9 glucose to ampicillin containing M9 with different carbon source media. **(C)** Comparison of persister level at 24 h after nutrient downshift from M9 glucose to antibiotic containing M9 with different carbon media. Statistical results in **(B,C)** were calculated using a one-way ANOVA method with Dunnett’s test of 24 h survival rates. For each antibiotic tested in **(C)**, the persister levels of GLU → MAL, GLU → PYR, GLU → FUM, and GLU → SUC were compared with GLU → OA. Statistical results indicating significant differences from GLU → OA were labeled. **(D)** Time-lapse images of cells during ampicillin treatment period after nutrient downshift from M9 glucose to oleic acid (top) and M9 fumarate (bottom).

Cells demonstrate remarkable metabolic plasticity, allowing them to efficiently use a wide range of metabolic substrates and adapt to changing environmental conditions. This adaptability is essential for their survival and sustained function (Cabodevilla et al., 2013). Following a GLU → OA + AMP switch, 56% of cells survived 24-h of ampicillin treatment (Hartline et al., 2022; Zhang et al., 2024). We first assessed whether this high persistence level is unique to the GLU → OA + AMP switch or can be triggered by a nutrient shift from glucose to other gluconeogenic carbon sources in the presence of ampicillin. We selected pyruvate (PYR), malate (MAL), fumarate (FUM), and succinate (SUC) for nutrient shift persister assays, as these carbons represent different entry points into glycolysis and the TCA cycle. Cells at designated time points after nutrient shift were collected, washed and plated on antibiotic-free plates to determine survivor counts and generate time-kill curves (Figure 1A). Unlike GLU → OA + AMP shift, glucose shifts to all four tested gluconeogenic carbons exhibited normal cell killing dynamics, with persistence levels ranging from 10^−6^ to10^−7^ (Figure 1B), consistent with wild-type (WT) *E. coli* persistence levels in exponentially growing cell cultures (Vázquez-Laslop et al., 2006). Additionally, these shifts did not induce notable transient tolerance, suggesting *E. coli* resumed growth quickly after switching without the extended lag phase as observed during a shift to OA (Supplementary Figure S1).

To test whether the elevated persistence level after GLU → OA + AMP switch is specific to ampicillin, we performed the nutrient shifts and time-kill experiments using an alternative beta-lactam antibiotic, carbenicillin (CRB), or an aminoglycoside, gentamicin (GEN). For both CRB and GEN, high levels of persistence (>1% persisters at 24 h post-shift) were only observed after GLU → OA + ABX shift, not with other gluconeogenic carbon shifts (Figure 1C), indicating this effect is specific to the GLU → OA + ABX condition.

The unique high persistence makes the GLU → OA + ABX shifts a compelling model to study how metabolism drives persister formation. We focused on ampicillin due to its characteristic lysis-based killing, which can be readily visualized under the microscope. Time-lapse microscopy was used to monitor cell morphological changes post nutrient shifts. After shifting from glucose to pyruvate, succinate, malate, or fumarate, cells lysed in the presence of ampicillin as indicated by sudden disruptions in cell outlines (Supplementary Videos S1–S4). Cell lysis typically occurred within 1–4 h post-shift, consistent with the time-kill data. Cell lysis in the presence of ampicillin is also consistent with the primary killing mechanism: inhibition of peptidoglycan biosynthesis weakens bacterial cell wall, making it unable to withstand the internal pressure (Kawai et al., 2023). In contrast, cells after the GLU → OA + AMP shift remained intact without lysis throughout the 24-h ampicillin treatment, indicating ampicillin failed to weaken bacterial cell walls (Figure 1D; Supplementary Video S5).

### Glucose-to-oleic acid downshift triggers ppGpp-dependent persistence

Glucose is the preferred carbon source for *E. coli*. Previous studies have shown that shifting cells from a preferred to a less preferred carbon source can trigger the stringent response, mediated by the signaling molecules guanosine tetra- and penta-phosphate, collectively known as (p)ppGpp (referred to here as ppGpp) (Ahn-Horst et al., 2022; Zhu and Dai, 2023). Accumulation of ppGpp downregulates multiple growth-related processes and upregulates stress response pathways. Under extreme conditions, ppGpp promotes dormancy and enhances persistence level (Amato et al., 2013; Kotte et al., 2014; Amato and Brynildsen, 2015; Radzikowski et al., 2016). Given this role, we investigated whether the high persistence observed after the GLU → OA + AMP downshift is associated with elevated ppGpp level.

We used an RNA-based ppGpp biosensor developed by Sun et al. (2021) to measure ppGpp levels in single cells following nutrient shifts. The biosensor comprises a ppGpp-specific aptamer, a transducer sequence, and a fluorogenic RNA reporter. Upon ppGpp binding, the transducer promotes hybridization that enables the RNA reporter to bind its cognate dye, DFHBI-1T, activating fluorescence. We transformed *E. coli* with the ppGpp biosensor and kept the RNA constitutively transcribed while cultivating the cells in either a steady state growing phase in M9 glucose media or subjected to nutrient shifts to M9 OA media supplemented with ampicillin.

**Figure 2:**
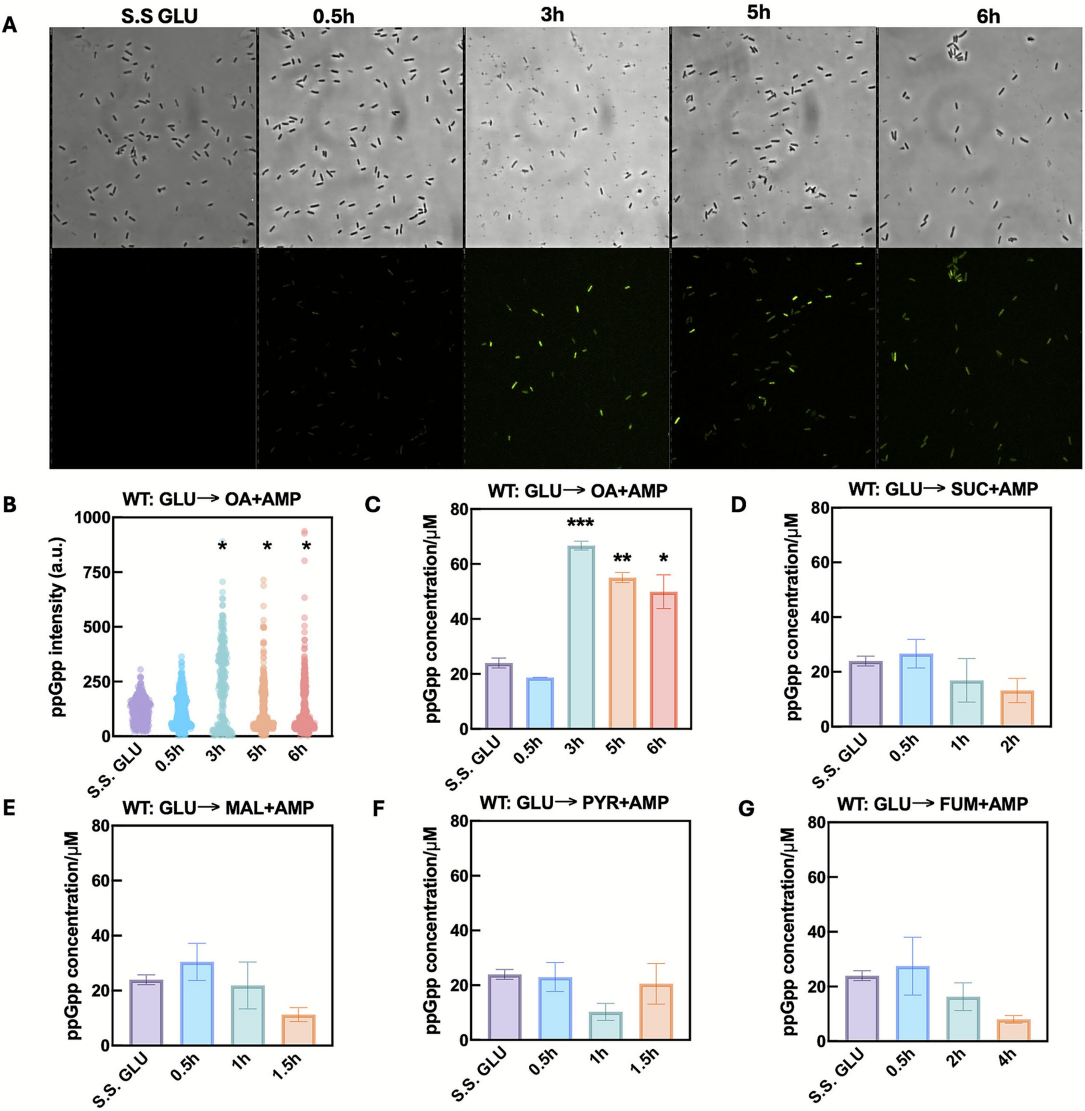
Glucose-to-oleic acid downshift triggers ppGpp-dependent persistence. **(A)** Microscopy images of cells carrying the RNA-based ppGpp biosensor at different stages during a glucose to ampicillin containing oleic acid shift. **(B)** Relative ppGpp intensity in single cells (*n*>300 from biological triplicates) at different time points after the nutrient shift from glucose to ampicillin-containing oleic acid. *p* values were calculated using a pairwise comparison method with Holm-Sidak’s test. Intracellular ppGpp level quantified with HPLC at different time points of nutrient downshift from glucose to ampicillin-containing oleic acid **(C)**, succinate **(D)**, malate **(E)**, pyruvate **(F)**, and fumarate **(G)**. Statistical results in **(C–G)** were calculated using a one-way ANOVA method with Dunnett’s test.

Cells grown on glucose displayed weak green fluorescence, establishing the baseline ppGpp level (Figure 2A). At 30 min after the GLU → OA + AMP shift, fluorescence was slightly higher but not statistically different from the glucose control. However, by 3 h, a subpopulation of cells exhibited a substantial increase in fluorescence intensity and lasted through 6 h post-shift, indicating elevated ppGpp levels (Figures 2A,B). This increase coincided with the transient tolerance period observed in earlier persistence assays (Figure 1B) and is likely due to impaired FA utilization, potentially caused by limited FA degradation enzymes (Zhang et al., 2024). The presence of high-fluorescence cells supports a potential role for ppGpp in persistence formation.

To further confirm ppGpp accumulation dynamics, we also quantified the population-average intracellular ppGpp levels using HPLC, following published methods (Radzikowski et al., 2016). The standard curve and chromatograms are presented in Supplementary Figure S2. The population level ppGpp change closely mirrored biosensor results (Figure 2C), showing an approximately 3-fold increase at 3 h followed by a slight decline at 6 h. In contrast, no significant ppGpp elevation was observed after shifting glucose to other gluconeogenic carbon sources (Figures 2D–G). Instead, ppGpp levels steadily declined, likely due to cell death post-shift. Collectively, these findings confirm that ppGpp accumulates following the GLU → OA + AMP shift. Its accumulation kinetics and heterogeneity exhibiting a striking resemblance to the observations from time-kill survival kinetics.

### ppGpp mediates higher persistence via inhibiting phospholipid synthesis

We next aimed to dissect the molecular mechanisms linking ppGpp to persistence during the GLU → OA + AMP shift. Previous studies have shown that ppGpp inhibits bacterial growth-related processes in a concentration-dependent and sequential manner, with different pathways requiring distinct ppGpp levels for inhibition (Balsalobre, 2011; Steinchen et al., 2020; Czech et al., 2022). Thus, the specific ppGpp-triggered growth-inhibition mechanism may be condition-dependent.

**Figure 3:**
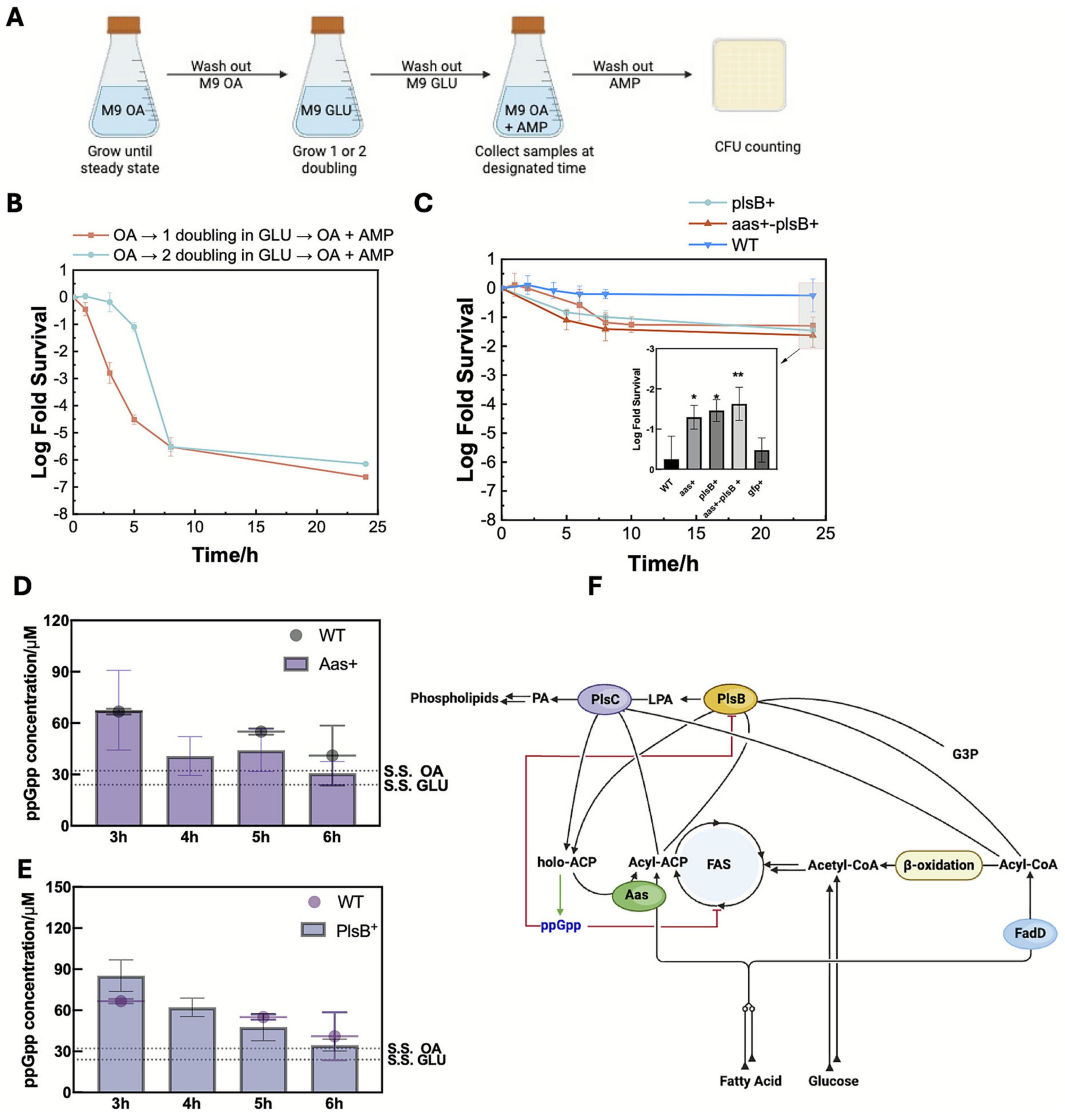
ppGpp mediates higher persistence via inhibiting phospholipid synthesis. **(A)** Nutrient shift persister experimental procedure of reducing ppGpp accumulation time by adjusting pre-shift FadD level. **(B)** Time–kill curves of different pre-shift FadD levels after a glucose to ampicillin containing oleic acid shift. **(C)** Persister level of overexpression strains at 24 h after a glucose to ampicillin containing oleic acid shift. Statistical results in **(B,C)** were calculated using a one-way ANOVA method with Dunnett’s test of 24 h survival rates. Intracellular ppGpp level quantified with HPLC at different time points of nutrient downshift from glucose to ampicillin-containing oleic acid of *aas^+^* **(D)** and *plsB^+^* **(E)** mutant strain. **(F)** Proposed ppGpp-dependent persister formation mechanism following the nutrient shift from glucose to oleic acid in the presence of ampicillin. Statistical results in **(D,E)** were calculated using a one-way ANOVA method with Dunnett’s test.

We first hypothesized that the elevated ppGpp level was triggered by carbon starvation. When growing *E. coli* in glucose media, FA degradation pathway is tightly repressed by catabolic repression. After the GLU → OA + AMP shift, cells require up to 6 h to fully express FA degradation enzymes (Zhang et al., 2024), creating an extended starvation period during the lag phase. This prolonged starvation does not occur when cells are shifted from glucose to other gluconeogenic carbons (Supplementary Figure S1). We hypothesized that shortening the starvation period might reduce ppGpp-triggered persister formation. To test this, cells were pre-grown in M9 OA media to fully induce FA degradation enzymes, then transferred to M9 glucose for 1 or 2 generations, corresponding to 1/2 or 1/4 dilution of FA degradation enzyme levels. We then performed the GLU → OA + AMP nutrient shift and time-kill assay (Figure 3A). The results revealed that cells grown in glucose for 1 generation retained sufficient FA degradation capacities to immediately metabolize OA, resulting in rapid killing of 99.99% of cells (Figure 3B). In contrast, cells grown in glucose for two generations exhibited a up to 3-h transient tolerance period following the shift due to insufficient initial FA degradation enzymes. FA catabolism subsequently resumed, leading to the death of 99.99% of cells. High persistence was only observed when the lag phase was long enough to induce carbon starvation, consistent with the ppGpp accumulation kinetics (Figures 2C–G).

We next hypothesized that FA starvation drives ppGpp accumulation through holo-ACP. After the GLU → OA + AMP shift, FA degradation is activated while FA biosynthesis is repressed by FadR and FabR (My et al., 2015). Repression of FA biosynthesis depletes acyl-ACP and holo-ACP pool size relatively increases. Unacylated holo-ACP then activates the ppGpp synthase activity of SpoT, while reducing its hydrolase activity (Battesti and Bouveret, 2006; Angelini et al., 2012; Fernández-Coll and Cashel, 2020), thereby driving ppGpp accumulation. Elevated ppGpp further amplifies the effect by inhibiting FA biosynthesis enzymes, thus forming a positive feedback loop. To test this, we overexpressed the *E. coli* acyl-ACP synthetase (encoded by *aas*), which ligates free FAs to holo-ACP, regenerating acyl-ACP (Jackowski et al., 1994). We performed GLU → OA + AMP shift and time-kill assays using the *aas* overexpression strain (Figure 3C, *aas^+^*), and quantified the ppGpp concentration with HPLC. *Aas*^+^ mutant strain showed a trend of reduced ppGpp accumulation at 3 h and, more notably, failed to sustain the elevated level seen in the WT at later time points, consistent with acyl-ACP synthetase restoring acyl-ACP as imported FAs accumulated overtime (Figure 3D) (Jackowski et al., 1994). This reduced ppGpp allowed growth to resume, leading to rapid antibiotic killing. Consistently, the time-kill assay revealed a substantially reduced persistence observed in the *aas^+^* strain, with 95% cells killed after 24 h, compared to 44% in the wildtype strain. As a negative control, overexpression of green fluorescent protein (GFP) from the same promoter showed no effect on persistence (Figure 3C, *gfp*^+^), ruling out the possibility of vector and protein overexpression-induced differences. We further hypothesized that ppGpp promotes persister formation in wildtype *E. coli* by inhibiting PlsB, the sn-glycerol-3-phosphate acyltransferase that catalyzes the first committed step of phospholipid biosynthesis (Heath et al., 1994; Yao and Rock, 2013). To test so, we constructed a *plsB* overexpression mutant (*plsB^+^*) to alleviate ppGpp inhibition through increasing the enzyme copy number. We found that the *plsB^+^* strain displayed reduced persistence, with 97% cells killed after 24 h in OA + AMP (Figure 3C). HPLC results showed that ppGpp kinetics in *plsB+* (Figure 3E) largely matched with that in wildtype. Thus, excess PlsB likely enabled continued phospholipid synthesis despite ppGpp, accelerating recovery and antibiotic killing. Finally, overexpressing both *aas* and *plsB* (*aas-plsB*^+^) did not further decrease persistence beyond single overexpression (~97.6% killed, Figure 3C). This lack of synergy is likely due to the metabolic coupling: PlsB consumes acyl-ACP, the product of acyl-ACP synthetase, regenerating holo-ACP and offsetting the effect on ppGpp (Figure 3F).

### Metabolism changes in persisters formed after GLU → OA + AMP nutrient downshift

**Figure 4:**
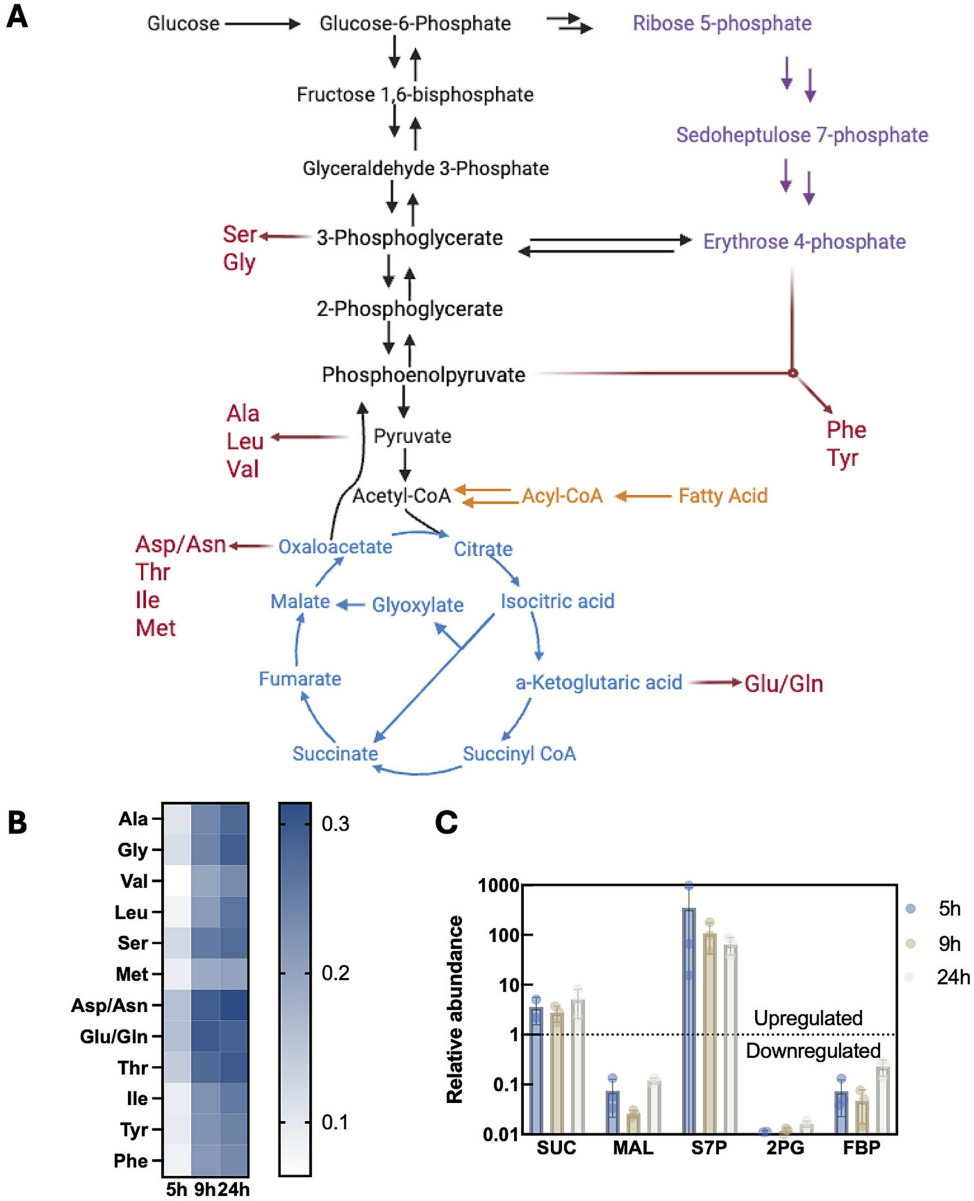
Bacterial persister metabolism during nutrient downshift from glucose to oleic acid. **(A)** Metabolic pathways involved in nutrient downshift from glucose to ampicillin-containing oleic acid shift. **(B)** ^13^C labeled proteinogenic amino acid enrichment at 5 h, 9 h, and 24 h after nutrient shift from ^12^C-glucose to fully labeled ^13^C -oleic acid along with ampicillin. **(C)** Key metabolite relative abundance after nutrient shift from glucose to ampicillin-containing oleic acid to that of cells exponentially growing on glucose. SUC, Succinate; MAL, Malate; S7P, Sedoheptulose 7-phosphate; 2PG, 2-Phosphoglycerate; FBP, Fructose 1,6-biphosphate.

At last, we sought to understand whether persisters formed after the GLU → OA + AMP shift remains metabolic activity or enter complete dormancy. To examine amino acid and protein synthesis, we performed GLU → OA + AMP shift using unlabeled (^12^C) glucose and fully ^13^C-labeled OA. Cells were harvested at 5, 9, and 24 h post-shift, with 5 h representing the end of the transient tolerance phase and the last two time points within the persistence window. Total proteins were extracted, hydrolyzed into amino acids, and analyzed via GC-MS (Figure 4B). At 5 h post-shift, ^13^C incorporation into proteinogenic amino acids was minimal, indicating very low flux into amino acid biosynthesis, consistent with the lack of FA catabolism due to insufficient β-oxidation enzymes. By 9 h, elevated ^13^C enrichment was detected across all measured amino acids. These results demonstrates that carbon derived from β-oxidation entered central pathways for amino acid and protein biosynthesis, confirming that persisters remained metabolically active rather than fully dormant during persistence (Blattman et al., 2024; Fung et al., 2025). The ^13^C incorporation involves β-oxidation to acetyl-CoA, the TCA cycle, gluconeogenesis, and the pentose phosphate pathway (e.g., valine and histidine, Figure 4A). Additional ^13^C incorporation continued beyond 9 h, but at a slower rate, reflecting emerging metabolic bottlenecks despite substrate availability.

To further examine metabolic activity, we quantified intracellular metabolite pools using LC–MS and compared them to exponentially growing glucose culture (Figure 4C). Substantial changes were observed across key metabolites, marked by the depletion of malate, 2-phosphoglycerate (2PG), and fructose 1,6-bisphosphate (FBP), alongside the accumulation of succinate and sedoheptulose 7-phosphate (S7P). This signature reflects a defined metabolic strategy to cope with stress and indicates potential bottlenecks for growth resumption.

The depletion of FBP and 2PG confirms switch away from glucose catabolism and relief of inhibition on gluconeogenesis, allowing reverse flux from TCA intermediate up to gluconeogenesis. The sharp reduction in malate, despite abundant succinate, suggests its rapid conversion to oxaloacetate or pyruvate. Meanwhile, the significantly elevated S7P abundance indicates an active pentose phosphate pathway (PPP) activity. This metabolic shift represents a key adaptive response, as ampicillin treatment induces oxidative stress as part of lethality (Dwyer et al., 2014; Zhang et al., 2024). To survive, persister cells must detoxify reactive oxygen species (ROS) and maintain essential redox homeostasis. Channeling carbon flux through the PPP serves as a critical survival strategy to meet this demand, as it generates the NADPH required for antioxidant defense and redox balance under antibiotic pressure. The accumulation of S7P also suggests a persister state, as ppGpp shuts down the expensive DNA and RNA synthesis, which reduces the demand for nucleotides building block, ribose 5-phosphate (R5P), and as a result, leads to the accumulation of S7P. This metabolic reprogramming trend is consistent with the ppGpp-dependent transcriptional response profile observed during amino acid starvation (Traxler et al., 2008). Together, these results show that persisters after the GLU → OA + AMP shift undergo active carbon flux reprogramming rather than metabolic shutdown. Activation of gluconeogenesis and the pentose phosphate pathway provide a survival strategy to balance redox homeostasis and counter oxidative stress, thereby enabling persistence and evasion of β-lactam killing (Kawai et al., 2019).

## Discussion

This work identifies the GLU → OA nutrient shift as a unique trigger of *E. coli* persistence against β-lactams and aminoglycosides. The central determinant of this phenomenon is ppGpp. Real-time biosensing and HPLC quantification both revealed that ppGpp rises sharply during the prolonged lag phase that follows the GLU → OA + AMP switch, but not during other nutrient shifts. Manipulations that shorten starvation or lower ppGpp accumulation via acyl-ACP synthase overexpression reduced persistence, directly linking FA starvation and elevated ppGpp level with antibiotic persistence. Mechanistically, our results suggest that following the GLU → OA + AMP shift, FA catabolism represses FA biosynthesis, depleting acyl-ACP, which in turn stimulates SpoT-mediated ppGpp synthesis. Elevated ppGpp can inhibit transcription of *fabH* and other FA biosynthesis genes (43, 44), while also suppressing phospholipid synthesis through inhibition of PlsB enzyme activity. The combined effect halts cell growth and promotes antibiotic tolerance. Overexpression of *aas* reduced ppGpp levels while overexpression of *plsB* alleviate the inhibition of membrane biosynthesis, both leading to reduced persistence and supporting the central role of FA–ppGpp coupling in persister formation.

Stable isotope tracing and metabolomics further demonstrate that persisters triggered by GLU → OA + AMP shift are not metabolically inert. Instead, they actively channel carbon from FA through β-oxidation, the TCA cycle, gluconeogenesis, and the pentose phosphate pathway. This flux reprogramming sustains redox balance, supports limited biosynthesis, and helps cells withstand ROS. These observations highlight persistence as a metabolically adaptive, rather than dormant state, under our experimental conditions.

These findings contribute to our understanding of bacterial persistence. They show that metabolic shift can program persistence through defined regulatory and metabolic pathways (Schmitz et al., 2017; Evans and Zhang, 2020). By revealing how nutrient downshifts couple FA metabolism, ppGpp signaling, and redox-protective fluxes, this work highlights new vulnerabilities: targeting ppGpp synthesis indirectly via acyl-ACP balance or forcing metabolic bottlenecks open with metabolite supplementation may collapse persistence and restore antibiotic efficacy. More broadly, our study illustrates how environmental nutrient transitions, prevalent in host niches, can remodel bacterial physiology to promote survival under antibiotic stress. Given that many molecular pathways regulating stress responses, toxin-antitoxin systems, metabolism, and survival are conserved among bacteria, this insight not only deepens the mechanistic understanding of persistence but also reinforces metabolic interventions as a new front in the fight against recurrent and recalcitrant infections (Allison et al., 2011b).

## Data Availability

The datasets presented in this study can be found in online repositories. The names of the repository/repositories and accession number(s) can be found in the article/Supplementary material.
