# Supplementary material for: (p)ppGpp mediates persister formation in Escherichia coli during glucose to fatty acid shift
Source: Front Microbiol. 2026 Jan 16;16:1749456. doi: 10.3389/fmicb.2025.1749456 (PMC12858184; doi:10.3389/fmicb.2025.1749456)
Supplement: Supplementary file 7 [file Data_Sheet_2.DOCX]

**(p)ppGpp Mediates Persister Formation in *Escherichia coli* During Glucose to Fatty Acid Shift**

Ruixue Zhang ^a^, Zhengyang Xiao ^a^, Neha Namburi ^a^, Yinjie Tang ^a^, Joshua Yuan ^a^, Fuzhong Zhang ^a,b,c,*^

a Department of Energy, Environmental & Chemical Engineering, Washington University in St. Louis, St. Louis, Missouri, USA

b Division of Biological and Biomedical Sciences, Washington University in St. Louis, Saint Louis, Missouri, USA

c Institute of Materials Science and Engineering, Washington University in St. Louis, Saint Louis, Missouri, USA

*Address correspondence to Dr. Fuzhong Zhang, Email: fzhang@seas.wustl.edu; Tel: 314-935-7671; FAX: 314-935-7211

Running title: (p)ppGpp Mediates Nutrient-Shift Persister Formation

**Video 1** 6-hour Time-lapse video of *E. coli* cells after a nutrient downshift from glucose to ampicillin-containing M9 pyruvate agarose pad

**Video 2** 6-hour Time-lapse video of *E. coli* cells after a nutrient downshift from glucose to ampicillin-containing M9 succinate agarose pad

**Video 3** 6-hour Time-lapse video of *E. coli* cells after a nutrient downshift from glucose to ampicillin-containing M9 malate agarose pad

**Video 4** 6-hour Time-lapse video of *E. coli* cells after a nutrient downshift from glucose to ampicillin-containing M9 fumarate agarose pad

**Video 5** 24-hour Time-lapse video of *E. coli* cells after a nutrient downshift from glucose to ampicillin-containing M9 oleic acid agarose pad

**Figure S1** A) Lag time profiles after nutrient shifts from M9 minimal glucose to different carbons in the absence of ampicillin, statistical significance was determined using unpaired Welch's t-tests performed on group means and standard deviations (n=3 biological replicates). B) Wildtype NCM3722 *E. coli* cell growth curves after nutrient shift from M9 minimal glucose to various carbons in the absence of ampicillin.

A

B


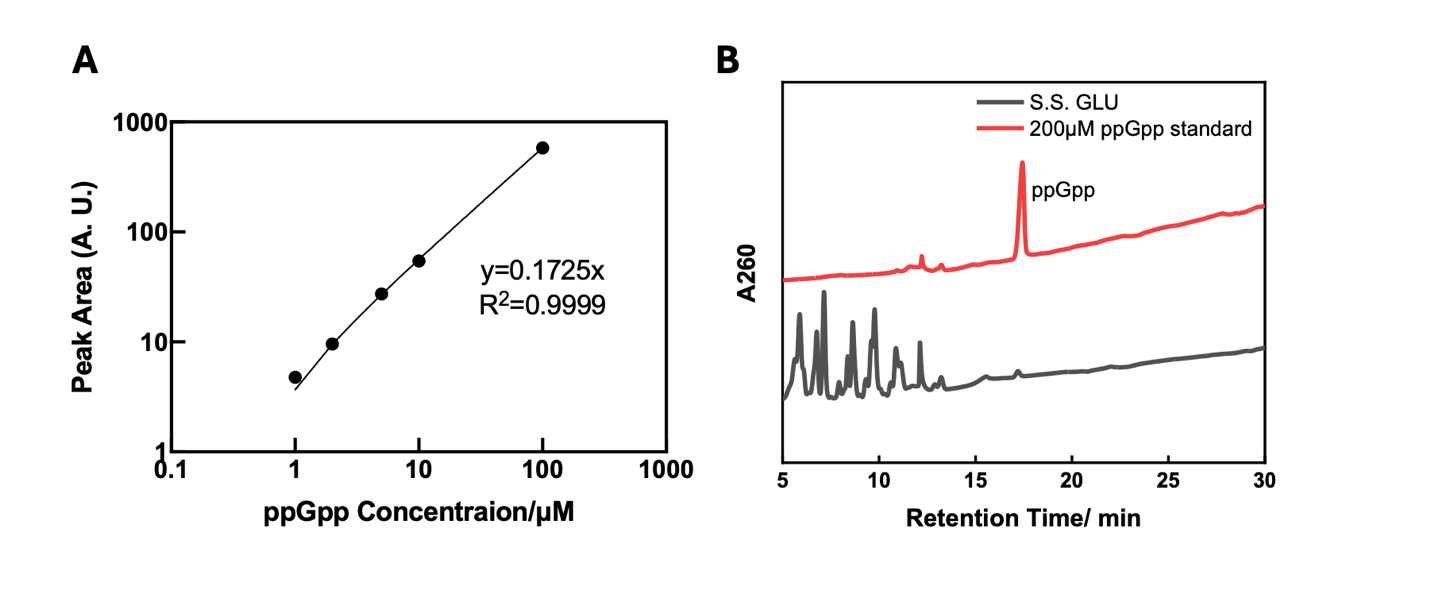


Figure S2 A) ppGpp HPLC quantification standard curve. B) Chromatograms of 200μM ppGpp standard sample and steady state cells growing in M9 glucose sample.

**Table S1 Plasmids**

| **Plasmid** | **Function** | | **Source** |
| --- | --- | --- | --- |
| pTarget_T7RNAP | | Used for knock-in of T7-RNAP to NCM3722 genome at ybhC locus | This work |
| pCas | | Constitutive expression of cas9 and inducible expression of lambda RED and sgR | Addgene(#62225) (Jiang et al., 2015) |
| pET-28c S2 | | Used for ppGpp imaging | Addgene (#171920) (Sun et al., 2021) |
| Ps6c-aas | | Used for aas overexpression | This work |
| Ps6c-plsb | | Used for plsB overexpression | This work |
| Ps6c-aas-plsB | | Used for aas and plsB overexpression | This work |
| Ps6c-gfp | | Used for gfp overexpression | (Zhang et al., 2024) |

**Table S2 Strains**

| **Strain** | | **Genotype** | **Source or reference** |
| --- | --- | --- | --- |
| NCM3722 (WT) | F^+^ | | CGSC#:12355 |
| MDS42pdu | MDS42 with polB， dinB， umuDC deleted | | (Csörgő et al., 2012) |
| NCM3722 (DE3) with ppGpp sensor | NCM3722 ybhC::T7 RNAP with pET-28c S2 | | This work |
| NCM3722 *aas*^+^ | NCM3722 with ps6c-aas | | This work |
| NCM3722 *plsB*^+^ | NCM3722 with ps6c-plsB | | This work |
| NCM3722 *aas*^+^- *plsB*^+^ | NCM3722 with ps6c-aas-plsB | | This work |

**References**

Csörgő, B., Fehér, T., Tímár, E., Blattner, F. R., and Pósfai, G. (2012). Low-mutation-rate, reduced-genome Escherichia coli: an improved host for faithful maintenance of engineered genetic constructs. *Microb Cell Fact* 11, 11. doi: 10.1186/1475-2859-11-11

Jiang, Y., Chen, B., Duan, C., Sun, B., Yang, J., and Yang, S. (2015). Multigene Editing in the Escherichia coli Genome via the CRISPR-Cas9 System. *Appl Environ Microbiol* 81, 2506–2514. doi: 10.1128/AEM.04023-14

Sun, Z., Wu, R., Zhao, B., Zeinert, R., Chien, P., and You, M. (2021). Live‐Cell Imaging of Guanosine Tetra‐ and Pentaphosphate (p)ppGpp with RNA‐based Fluorescent Sensors**. *Angew Chem Int Ed* 60, 24070–24074. doi: 10.1002/anie.202111170

Zhang, R., Hartline, C., and Zhang, F. (2024). The ability in managing reactive oxygen species affects Escherichia coli persistence to ampicillin after nutrient shifts. *mSystems* 9, e01295-24. doi: 10.1128/msystems.01295-24
